# Draft Genome Sequence of *Bacillus megaterium* BHG1.1, a Strain Isolated from Bar-Headed Goose (*Anser indicus*) Feces on the Qinghai-Tibet Plateau

**DOI:** 10.1128/genomeA.00317-16

**Published:** 2016-05-12

**Authors:** Wen Wang, Si-Si Zheng, Hao Sun, Jian Cao, Fang Yang, Xue-Lian Wang, Lai-Xing Li

**Affiliations:** a Key Laboratory of Adaptation and Evolution of Plateau Biota, Northwest Institute of Plateau Biology, Chinese Academy of Sciences, Xi’ning, China; b Center of Growth, Metabolism and Aging, College of Life Sciences, Sichuan University, Chengdu, China; c University of the Chinese Academy of Sciences, Beijing, China

## Abstract

*Bacillus megaterium* is a soil-inhabiting Gram-positive bacterium that is routinely used in industrial applications for recombinant protein production and bioremediation. Studies involving *Bacillus megaterium* isolated from waterfowl are scarce. Here, we report a 6.26-Mbp draft genome sequence of *Bacillus megaterium* BHG1.1, which was isolated from feces of a bar-headed goose.

## GENOME ANNOUNCEMENT

*Bacillus megaterium* is a soil-inhabiting Gram-positive, spore-forming, and rod-shaped bacterium. This microbe was first described by Anton De Bary in 1884 (1). Due to its large cell size, *B. megaterium* is well suited for research on cell morphology, sporulation, and protein localization (2). It has also exhibited many advantages in the industrial applications for recombinant protein and vitamin production (3, 4), as well as for bioremediation (5). Beyond these traditional applications, *B. megaterium* is now gaining particular interest as probiotic supplements for use in animal feeds because of its heat stability and ability to survive the gastric barrier (6, 7). Our previous comparative study showed that the relative abundance of genus *Bacillus* was significantly higher in the gut microbiota of wild bar-headed geese compared to farmed ones (8, 9). Therefore, there is a great need to isolate some candidate probiotic strains of *Bacillus megaterium* from a wild bar-headed goose. In this direction, the work here presents the draft genome sequence of *Bacillus megaterium* BHG1.1, which was isolated from a pooled fecal sample collected from a wild bar-headed goose.

The whole-genome DNA was extracted and then sequenced using the Illumina HiSeq 4000 platform (one-lane, paired-end run [2 × 150 bp]). A total of 9,771,088 paired-end reads with lengths of 150 nucleotides of 1,465,663,200 bp were generated, which, after quality control, resulted in 9,270,858 clean reads of 1,366,096,694 bp. *De novo* assembly of these clean reads was carried out using the newly developed SPAdes algorithm version 3.6.2 (10) followed by error correction using the Bayes Hammer program (11). The reads were assembled into 92 contigs, with an *N*_50_ value of 1.2 Mb and the largest contig size of 2.1 Mb. Then, gene prediction was performed with Prodigal version 2.6.2 (12), while tRNA was predicted with tRNAScan-SE version 1.3.1 (13), and rRNA was predicted with RNAmmer version 1.2 (14). Subsequently, the gene functions were annotated into the KEGG (15) and COG (16) databases using BLASTx.

The genome is 6,263,758 bp long, which appears to be larger than other finished genomes of *B. megaterium*, with a G+C content of 37.20%. The most similar strain to strain BHG1.1 in databases is *B. megaterium* QM B1551 (GenBank accession no. NC_014019.1) with an average nucleotide identity (17) value of 96.76%. This microbe possesses 6,682 genes, 6,426 coding sequences (CDSs), 112 tRNAs, and 5 rRNAs. Approximately 60.29% (*n* = 3,874) of the CDSs were assigned to functional COG categories and 38.80% (*n* = 2,493) were assigned a KEGG orthology (KO) number. Based on KEGG pathway analysis, most genes encode proteins involved in metabolism and environmental information processing.

Further studies are under way to determine the probiotic potential of *B. megaterium* BHG1.1, including its ability to suppress the growth of pathogenic microbes and to productively influence the immune response.

### Nucleotide sequence accession numbers.

This whole-genome shotgun project has been deposited in GenBank under the accession number LUCO00000000. The version described in this paper is the first version, LUCO01000000.
